# Mesenchymal Stem Cells as a Prospective Therapy for the Diabetic Foot

**DOI:** 10.1155/2016/4612167

**Published:** 2016-10-27

**Authors:** Qinan Wu, Bing Chen, Ziwen Liang

**Affiliations:** Department of Endocrinology, The First Affiliated Hospital of The Third Military Medical University, Chongqing 400038, China

## Abstract

The diabetic foot is a serious complication of diabetes. Mesenchymal stem cells are an abundant source of stem cells which occupy a special position in cell therapies, and recent studies have suggested that mesenchymal stem cells can play essential roles in treatments for the diabetic foot. Here, we discuss the advances that have been made in mesenchymal stem cell treatments for this condition. The roles and functional mechanisms of mesenchymal stem cells in the diabetic foot are also summarized, and insights into current and future studies are presented.

## 1. Introduction

China has nearly 100 million adults with diabetes [1], and there are 386 million diabetes patients worldwide. If this number continues to increase at the current rate, it will be 558 million. The diabetic foot is the most serious complication of diabetes and the main cause of amputation without trauma. In particular, diabetes patients often have severe ischemic disease in the lower extremities, so the probability of these patients requiring an amputation is 40 times the rate in nondiabetic patients. In fact, approximately 85% of all amputations occur in diabetic patients, and approximately 15% of diabetic patients will experience diabetic foot ulcers during their lifetime. The diabetic foot accounts for 22% of the total cost of treating diabetes, and the morbidity and mortality associated with this condition are very high [2]. Traditional therapies, including offloading, therapy for infection and ischemia, and local therapy, do not change the fundamental pathology underlying the diabetic foot, and their therapeutic effects are not satisfactory. The development of vascular interventional technology has brought about new hope to practitioners who treat diabetic feet, as it is minimally invasive, has a quick and curative effect, possesses advantages during long-term follow-up, and provides stability to the patient. This technology is widely used for the clinical treatment of diabetic feet, but for serious microvascular and macrovascular and nervous disorders of the diabetic vasculature, its circulation-improving effect is limited. Moreover, during long-term follow-up, the rates of restenosis and recovery of the peripheral circulation are still poor, and the amputation rate in patients with diabetic feet remains high. The diabetic foot is therefore a worldwide threat to public health [2].

## 2. The Application of Stem Cells in the Treatment of the Diabetic Foot: Stem Cell Therapy versus Cell Therapy

In recent years, the use of stem cell transplantation for the treatment of the diabetic foot has become widespread. This transplantation can promote the formation of new blood vessels, improve and restore limb blood flow, and improve ischemia [3]. It is thus also called cellular vascular bypass [4]. In 2002, Tateishi-Yuyama et al. treated lower extremity ischemic disease using stem cell transplantation and obtained optimal results. The mechanism of this treatment is believed to involve stem cells differentiating into vascular endothelial cells (VECs) and smooth muscle cells (SMCs) and the secretion of many angiogenic factors. In this process, the cells gradually differentiate, leading to the formation of new capillaries that can participate in vascular reconstruction to compensate for ischemia, in turn improving and restoring blood flow to the lower extremity [3].

At present, the main sources of stem cells that can be used in transplantation include bone marrow, peripheral blood, cord blood, umbilical cord tissues, and fat. According to the source, we can differentiate the cells into mononuclear cells (MNCs), endothelial progenitor cells (EPCs), or mesenchymal stem cells (MSCs). Among these, MSCs, which are derived from early mesoderm and ectoderm, have been widely investigated, especially in studies of tissue injury and repair. MSCs were first discovered by Friedenstein [5]. These cells have the potential to differentiate into osteoblasts, cartilage cells, fat cells, muscle cells, and nerve cells. Because they can differentiate into a diversity of cell types and because there are techniques available to isolate and amplify the numbers of these cells* in vitro*, MSCs are an ideal seed cell type for tissue engineering. These cells are most abundant in bone marrow, but they are also present in adipose tissue, cartilage, bone tissue, umbilical cord blood, and placentae [6, 7]. According to the criteria of the International Society for Cellular Therapy, MSCs exhibit adherent growth and the following phenotypes: 95% of the cells express CD105, CD73, and CD90, and most of the cells do not express CD45, CD34, and CD14 or CD11b, CD79a, and CD19. Moreover, these cells do not express MHC class II molecules, such as HLA antigens, and they have the potential to differentiate into osteoblasts, chondrocytes, adipocytes, and other types of cells [8]. In addition, a recent study found that MSCs play an anti-inflammatory role in immune regulation, whereby they can inhibit the proliferation of immune cells [9]. Here, we review the progress that has been made in MSC transplantation technologies for the treatment of diabetic lower limb ischemia, diabetic ulcers, and diabetic peripheral neuropathy in both animal models and clinical research.

## 3. Mesenchymal Stem Cell Transplantation and Diabetic Peripheral Artery Disease

### 3.1. Bone Marrow Mesenchymal Stem Cell versus Bone Marrow Mononuclear Cell Transplantation

Diabetes is an independent risk factor for peripheral vascular disease. Approximately 80 million diabetic patients in the United States have peripheral vascular disease, and 6.9–23.8% of diabetic patients over the age of 50 years have peripheral vascular disease in China. Critical limb ischemia in diabetic patients leads to a poor prognosis: the 5-year survival rate in these patients is only 50%, and mortality after amputation is 25–50%. Therefore, in patients with diabetes mellitus with peripheral artery disease (PAD), limb salvage is very important. Although the development of surgical techniques and interventional technologies may be beneficial in these patients, in 40% of these patients, peripheral vascular interventional and surgical indications and medications have no effect, which has resulted in the fact that there are no options for the treatment of these patients. In this subset of patients, stem cell therapy has shown a unique level of superiority [10, 11].

The cardiovascular research center of the Kansai Medical University first developed autologous bone marrow MNC (BMMNC) transplantation to treat lower limb ischemia in 2002. Since then, many countries have gradually applied bone marrow and peripheral blood MNCs in treating the diabetic foot in the clinical setting, with achievement of certain therapeutic effects. This technique can effectively improve the local blood supply and promote microcirculation reconstruction and wound healing [3, 4]. The limitations of BMMNC transplantation are that it has a more complex duration and a high amount of risk and is performed using a limited number of cells. Pittenger et al. revealed that bone marrow MSCs (BMMSCs) can differentiate into many types of cells and that they can reconstruct the microcirculation [12]. Al-Khaldi et al. found that blood flow in the chronic ischemic limb was improved after the transplantation of BMMSCs [13] and that BMMSCs could be used for skin wound healing in diabetic patients and mice [14, 15]. In another study, a total of 6 patients with diabetic feet and a level of Fontaine IV that had received no other treatment were transplanted with BMMSCs. The results showed that, after 6 months of follow-up, all of the patients had better blood flow reconstruction and wound healing, and the average healing time in 5 patients was nine months (1 patient died of heart failure). After five years of follow-up, the five patients showed good tissue healing and no adverse reactions, indicating that BMMSC transplantation is a potentially effective method for treating lower limb ischemia in patients with diabetes [16]. Procházka and colleagues used bone marrow mesenchymal cell transplantation in patients with diabetic feet and lower extremity vascular lesions who had experienced failed reconstructive vascular surgery. In these patients, the treatment significantly improved the toe brachial index (TBI), the percutaneous oxygen partial pressure, and the limb salvage rate by approximately 81% [17]. A total of 24 patients were followed up for 12 weeks, and the results suggested that treatment with BMMSCs significantly improved the pain-free walking distance and wound healing in diabetic patients with lower extremity vascular occlusive disease [18]. Lu and colleagues found that, after 24 weeks of follow-up, there was a significant difference between the effects of transplantation of autologous BMMNCs and transplantation of BMMSCs in terms of the amputation rate and the incidence of serious adverse events. However, when autologous BMMSCs were amplified and identified* in vitro* and then transplanted into patients to treat the diabetic foot, the patients showed improvements in the ankle-brachial index, the walking distance, and the number of angiographic collateral vessels on MRI [19]. In that study, the authors also found that transfection of peroxisome proliferator-activated receptor-*γ* coactivator-1 (PGC-1) into BMMSCs further increased angiogenesis, reduced apoptosis in endothelial cells, and improved the blood supply to ischemic lower limbs in an animal model. These data show that PGC-1 may be a target for treatments for the diabetic foot in patients with lower extremity vascular disease [20].

Animal experiments have shown that when bone marrow stem cells were prestimulated with G-CSF and then transplanted into rabbits with diabetic lower extremity vascular ischemia, the rabbits showed a higher level of expression of vascular endothelial growth factor (VEGF) and more angiogenesis more than in the control group, a bone marrow stem cell group, and a group transplanted with G-CSF-stimulated peripheral blood stem cells. In another animal study, BMMSCs were stimulated with epidermal growth factor, and the same results were obtained, indicating that this method may improve the efficiency of BMMSC transplantation. However, this technique can increase the risk of leukocytosis and a hypercoagulable state and can cause angina pectoris or acute arterial thrombosis [21, 22]. A study of 51 diabetic patients found that intramuscular transplantation of BMMSCs significantly improved the ankle-brachial index (ABI), the intermittent claudication distance, the transcutaneous oxygen partial pressure, and the rate of limb salvage compared to intramuscular transplantation of BMMNCs, suggesting that intramuscular BMMSC transplantation in patients with diabetic lower limb ischemia is a safe and effective treatment method compared to intramuscular BMMNC transplantation [23]. Researchers have shown that the survival rate in the case of BMMSC transplantation was increased by hypoxic preconditioning and that apoptosis was significantly decreased, while autophagy was increased. Incorporating the regulation of autophagy and hypoxic preconditioning may therefore provide new strategies for treating the diabetic foot with BMMSC transplantation [24].

In exploring the mechanisms that contribute to the effects of BMMSCs, researchers have found that BMMSCs secrete a variety of cytokines, such as SDF-1, VEGF, bFGF, and matrix metalloproteinases (MMPs), and that these cytokines promote the production of extracellular matrix (ECM) and reduce apoptosis in endothelial cells, as verified in models of endothelial dysfunction and endothelial promotion of vascular regeneration [25–30]. One study showed that BMMSCs increased the expression of angiopoietin-1 (Ang-1), which promoted the proliferation and migration of VECs [31]. Another study suggested that BMMSCs secrete a series of vascular growth factors which promote the formation of ECM to support the blood supply to arteries. Animal experiments have shown that BMMSCs promote the expression of collagen types I–IV in diabetic wound beds [32], secrete a series of growth factors and antifibrotic factors, including hepatocyte growth factor (HGF), interleukin-10 (IL-10), adrenomedullin [33], and MMP-9 [34, 35], and inhibit keratinocyte and fibroblast proliferation [36] and differentiation [37]. Other studies have shown that transplanted BMMSCs increase VEGF-A levels and the proliferation and migration of local endothelial cells via both paracrine and endocrine effects and that they promote the regeneration of blood vessels [38].

Research has suggested that intra-arterial transplantation of BMMSCs had a significantly better effect than intra-arterial transplantation of BMMNCs on diabetic lower limb arterial ischemia in terms of the ABI and wound healing [19]. In patients with arterial ischemia of the knee, BMMNC therapy is especially suitable, as intra-arterial perfusion of BMMNCs resulted in improvement in the Rutherford-Becker classification, the University of Texas diabetic wound scales, and the ABI in the target limb [39]. However, we think that BMMSCs may have a better effect, though this topic needs further exploration.

In summary, BMMSC transplantation has achieved positive results in treating diabetic lower extremity vascular ischemia in both animal experiments and human studies. The mechanism by which this therapy works involves both paracrine and endocrine effects by stem cells, which promote vascular endothelial regeneration and reconstruction of the microcirculation, improve endothelial dysfunction, and promote wound healing. At the same time, certain measures have been shown to improve the efficiency of cell transplantation and its treatment effects, including* in vitro* culture amplification, hypoxic preconditioning, growth factor prestimulation, promotion of cell autophagy, and overexpression of genes such as PGC-1. However, one study reported that transplantation of cultured mouse BMMSCs to treat diabetic neuropathy resulted in a 46% incidence of tumors at 4–8 weeks [40]. Therefore, the effectiveness and safety of these methods require further exploration.

### 3.2. Human Umbilical Cord Mesenchymal Stem Cell Transplantation

Umbilical cord blood is richer in stem cells than bone marrow, and because it is easy to obtain and has weak immunogenicity, it is viewed as a rich, low-cost, easy-to-collect source of stem cells [41]. The umbilical cord is the fetal connection to maternal and fetal cord-like structures, and it contains two umbilical arteries, one umbilical vein, and a tegumentary amniotic membrane. The arteriovenous component contains a special embryonic myxoid connective jelly-like tissue known as Wharton's jelly. Stem cells isolated from Wharton's jelly are referred to as human umbilical cord MSCs. Recently, a study found that fetal cells are stored in Wharton's jelly, which makes it a promising source of multipotent stem cells. In 2003, Mitchell et al. were the first to suggest that human umbilical cord stem cells have the characteristics of pluripotent stem cells, and a growing amount of research has shown that Wharton's jelly is a source of MSCs [42]. Wharton's jelly-derived mesenchymal cells contain MSCs, a class of immune cells which induce no immune rejection reaction, even in allogeneic transplantation. Many studies have suggested that these cells can significantly improve ischemia in peripheral vascular diseases in both nondiabetic patients and animal models.

One study showed that, at 1 week after angioplasty was performed using an interventional method, transplantation of human umbilical cord blood MSCs significantly improved symptoms in diabetic PAD patients with lower limb resting pain and an elevated skin temperature and promoted ulcer healing, even though new vessels had not yet formed. The mechanism for this effect may be the establishment of collateral circulation around the obstruction. At 1 month after the angioplasty, the ABI was significantly improved, and, at 3 months, visualization of the vasculature showed that the vascular vessel density was significantly increased, and the wound had healed well. The mechanism for this activity involves the proliferation and differentiation of stem cells into VECs and the secretion of a series of growth factors that promote angiogenesis and reconstruction of the collateral circulation to provide nutrition to obstructed distal vessels in the ischemic tissue. Even when there is proximal vascular restenosis, stem cells can still form a collateral blood supply to the distal vasculature, which is better than the results observed by traditional measurements, and these cells decrease the amputation rate and improve symptoms [43]. Interfering with fibroblast growth factor 4 (FGF4), stem cell factor (SCF), and the FLT3 ligand in cultures while amplifying umbilical cord blood MSCs* in vitro* can have endocrine and paracrine effects that lead to the differentiation of the cells into endothelial cells, improve diabetic ischemia in the lower limb by altering blood flow, and promote wound healing, with no obvious adverse reactions [44]. The intra-arterial transplantation of umbilical cord blood MSCs in rats with diabetic skin ulcers showed that the number of new blood vessels in the ulcer area significantly increased at the third day, that granulation tissue increased on the seventh day, and that stratified squamous epithelium was observed on the fourteenth day. The skin ulcer area was also significantly improved on the seventh and fourteenth days, and the mechanism proposed for this effect was promotion of the secretion of epithelial keratinocyte keratin 19 and participation in the formation of the ECM [45]. Another study found that stem cells that were isolated from Wharton's jelly and transplanted into diabetic mice with lower extremity vascular ligations significantly improved blood flow, increased capillary density, reduced apoptosis in endothelial cells, and increased the expression of hypoxia-inducible factor-1*α* (HIF-1*α*) and IL-8. Hypoxic preconditioning of Wharton's jelly in skeletal muscle cells led to a significant decrease in preapoptotic signaling proteins and an increase in antiapoptotic signaling proteins, and when HIF-1*α* and IL-8 were inhibited, these effects were attenuated; this finding suggests that HIF-1*α* and IL-8 play important roles in stem cell transplantation-based treatments for diabetic lower extremity vascular diseases [46].

The umbilical cord is richer in stem cells than the bone marrow is, and these cells are easy to collect and display weak immunogenicity [41]. Human umbilical cord MSCs are therefore a promising tool for effective treatment of diabetic lower limb ischemia.

### 3.3. Adipose Mesenchymal Stem Cell Transportation

In 2001, Zuk et al. isolated new adult stem cells for the first time. These cells were adipose-derived stem cells (ASCs), and the results of their study received a great deal of attention [47]. Because these cells are similar to BMMSCs, they are referred to as adipose MSCs. Since then, several research groups have used similar methods to isolate ASCs from adipose tissue. Because adipose tissue is easy to obtain, it is advantageous to use adipose tissue-derived MSCs instead of MSCs. According to previous studies, approximately 40 million ASCs can be obtained from each milliliter of adipose tissue, and a number of independent studies have shown that traditional extraction methods do not alter the activity of ASCs. Another study showed that ASCs and bone marrow-derived stem cells did not significantly differ in terms of several characteristics, including the rate at which cells were obtained, cell aging processes, gene transfection, and cell adhesion [48–50]. There are large numbers of stem cells in subcutaneous adipose tissue, and this tissue is easy to obtain without a painful procedure, such as bone marrow aspiration. Generally, when using local anesthesia, 100–200 mL of adipose tissue can be obtained. Studies have shown that an average gram of adipose tissue can produce 200000–290000 stem cells, meaning that 404000 stem cells can be separated per milliliter of fat. This tissue is thus a large and easily accessible source of stem cells for use in cell therapies [47, 48]. Other studies have found that 1 g of adipose tissue produces approximately 5 × 10^3^ stem cells, which is 500 times greater than the number of bone marrow stem cells produced per gram of bone marrow [49].

As a seed cell for transplantation, ASCs have been widely used during rehabilitation following plastic surgery, joint cartilage regeneration, repair of heart and brain function, and treatment of autoimmune and ischemic diseases. In Europe, ASCs have been used in clinical trials to treat chronic heart failure and acute myocardial infarction, with results suggesting that ASCs could be a valuable therapeutic option for vascular growth and tissue repair [47]. In addition, researchers have used ASCs as a carrier to deliver drugs to cells via a “homing” effect to inhibit the growth of mouse melanomas [49]. The mechanisms by which ASCs exert their effect in treatments for the diabetic foot include the following. (1) Many studies have suggested that ASCs have the ability to differentiate into endothelial cells and epithelial cells and can therefore improve the local blood supply to the wound and reconstruct the microcirculation [50–52]. (2) ASCs can accelerate the process of blood vessel construction and promote wound healing. In a study of ASC transplantation in pulmonary emphysema, ASCs were found to secrete an abundance of HGF, and, at 4 weeks after transplantation, the proliferation of epithelial cells and the number of blood vessels were increased, and pulmonary function was improved. The results further indicated that ASCs secrete vascular growth factor, which has a strong ability to promote angiogenesis in the local microenvironment and is involved in the revascularization process following tissue injury. Additionally, ASCs secrete several other cytokines and promote cell proliferation and wound healing. ASCs also secrete a large amount of HGF into the local microenvironment and participate in the revascularization of damaged tissue. ASCs can specifically differentiate into skin fibroblasts and keratin cells and act via paracrine and endocrine effects to promote skin repair and regeneration. In addition, ASCs secrete several cytokines to activate epidermal fibroblasts and keratin cells, and studies have shown that ASCs also secrete angiogenic factors, induce tissue regeneration, and participate in the process of immune cell regulation. However, the details of the mechanisms involved in chronic wound healing require further exploration [49–53].

In previous studies, transplanted adipose MSCs differentiated into endothelial cells and significantly improved blood flow and capillary density [54, 55] in a nude mouse model of ischemia, while transplantation of adipose-derived MSCs, which secrete VEGF and Von Willebrand factor, reduced endothelial cell apoptosis and promoted local microcirculation reconstruction [56, 57]. Animal experiments have shown that adipose MSCs increased the expression of sarcomeric actin, VEGF, and HGF when transplanted into rats with diabetic lower limb ischemia. MRI angiography also showed that the vascular density was increased and that the blood supply was significantly improved [58]. Jiang et al. found that overexpressing PGC-1*α* in adipose tissue-derived MSCs in an* in vitro* model of a diabetic environment reduced the increase in mitochondrial reactive oxygen species (ROS) which was induced by high glucose and hypoxia and decreased the rate of apoptosis in stem cells. These effects may improve the efficiency of transplantation [59]. In addition, research has revealed the following requirements of adipose MSC culture systems: −4°C, 20% albumin, 5% glucose Ringer's lactate fluid, and incubation for less than 48 h [60]. Three-dimensional culture technology can also be used to increase growth factor secretion in adipose MSCs in the liver and to improve the efficiency of transplantation [61].

In a clinical study, although adipose MSCs in diabetic patients had a reduced ability to proliferate compared to cells in nondiabetic people, after 6 months of intramuscular injections of autologous adipose MSCs, approximately 2/3 of patients with diabetic lower limb ischemia were in clinical remission (including improved resting pain and walking distance), and a significant increase in circulation was observed by angiography [62]. In a study of patients with nondiabetic lower limb ischemia who could not be treated via revascularization, adipose MSC transplantation improved their lower extremity percutaneous oxygen partial pressure and promoted local ulcer healing, showing that this technique is equally effective in patients with nondiabetic lower limb ischemia [63].

ASC research has mainly concentrated on* in vitro* and animal research, although many clinical trials in humans have been registered. However, most of these studies are still in progress, so their results have not yet been published. The problems in ASC research are mainly related to the following three facts: first, ASCs are a heterogeneous cell population, and specific markers for these cells have not been identified, which has become one of the key obstacles to future studies; second, there is no suitable animal model for culturing ASCs* in vitro* for transplantation, which is a common problem in animal experimental research; and, third, among human studies, there have been two case reports of thrombotic events after autologous adipose-derived MSC transplantation (NCT01257776). These events may be attributed to the fact that the autologous adipose tissue-derived MSC transplantation was performed in patients with diabetes mellitus. Although another study did not observe this complication (NCT00872326), it has been declared a safety problem in patients with diabetes [64].

Nevertheless, the sources of ASCs are convenient and rich in stem cells which display low immunogenicity. We therefore believe that ASCs will be widely favored by researchers as the seed cells for transplantation. With the rapid development of molecular biology, immunology, and cell biology techniques, research into ASCs will become more in-depth, the mechanisms by which ASCs improve the* in vivo* microenvironment will become a focus of research, and these factors will likely lead to major breakthroughs in which ASCs will become the ideal seed cells for tissue engineering research.

The results of the above studies suggest that different sources of stem cells for transplantation to promote angiogenesis in ischemic regions are available. In particular, MSCs can be differentiated into endothelial cells to promote angiogenesis in ischemic tissue. Currently, however, the mechanism by which MSCs promote angiogenesis is not clear. It is generally believed that MSCs can differentiate into VECs and SMCs, that they can directly form new blood vessels, and that they can participate in angiogenesis via factors including VEGF, basic fibroblast growth factor (bFGF), HGF, angiotensin-2, and angiotensin-1 [65]. Because there is potential for multiple differentiation events after MSC transplantation, the long-term safety and efficacy associated with this type of transplantation should be further explored.

## 4. Mesenchymal Stem Cell Transplantation for the Treatment of Diabetic Wounds

Wound healing is a complex process that involves five overlapping phases: coagulation, inflammation, migration, proliferation, and remodeling. Diabetic wound healing is an important problem, and the formation of wounds and peripheral vascular disease, trauma, infection, and neurological complications can overlap; the study of wound healing in this context therefore requires cooperation between many research areas [66]. Appropriate wound management is required and should include debridement, offloading, anti-infection treatments, blood circulation reconstruction, artery surgery, local dressing, and other measures [67]. However, these treatments are not satisfactorily effective [68], and there is therefore an urgent need to identify more practical approaches.

### 4.1. Bone Marrow Mesenchymal Stem Cells and Diabetic Wound Healing

In previous studies, researchers have suggested that the mechanism by which MSCs heal a wound is direct differentiation in wound tissues through a homing effect. In recent years, BMMSCs have been observed to display a sort of high self-renewal ability and to have the potential to differentiate into multiple lineages in addition to a homing effect. These cells can regulate inflammatory mediators and secrete growth factors to treat diabetic foot ulcers. In addition, the secretion of VEGF can stimulate the formation of granulation tissue and the migration of epithelial keratinocytes and fibroblast cells to promote ulcer healing. BMMSCs also have the potential to differentiate into epidermal cells and skin epithelial cells, which are involved in the formation of skin appendage structures and promote diabetic wound healing [32, 69–72]. Most of clinical studies have thus so far supported BMMSC transplantation to treat diabetic lower extremity vascular lesions and to promote the healing of ulcers [14, 18, 19]. Research has also shown that stem cells transplanted into the body can repair islet function in patients with islet dysfunction and can both improve blood glucose control and contribute to the healing of diabetic ulcers [73].

BMMSCs increase the secretion of body fluids, and cytokines play an important role in wound healing. MSCs specifically regulate immune responses and thereby participate in wound healing. For example, MSCs secrete immunoglobulin (Ig) M and IgG to combat inflammation in diabetic wounds. MSCs also inhibit macrophages from secreting a range of inflammatory cytokines, such as tumor necrosis factor-*α* (TNF-*α*), IL-6, and interferon-*α* (IFN-*α*), and stimulate the secretion of the inflammatory cytokines IL-10 and IL-12 to activate immune cells. Inflammation is thereby localized, which is conducive to wound healing [74, 75]. Furthermore, MSCs and cytotoxic T cells secrete inflammatory factors, including IL-10 and transforming growth factor-*β* (TGF-*β*), TDO 2,3-dioxygenase (IDO), nitric oxide (NO), and prostaglandin E2 (PGE2), and they reduce the local wound-induced inflammatory response. In addition, MSCs inhibit the activation of M1-type macrophages (inflammatory macrophages) and increase the activation of M2-type macrophages (anti-inflammatory macrophages). These cells can therefore have anti-inflammatory effects and promote the regression of chronic inflammation [76]. Studies have also shown that the expression of miR-146a is significantly decreased in diabetic wounds, and transplanted BMMSCs can significantly improve the availability of miR-146a, reduce the inflammatory state of the wound, and promote wound healing [77].

In addition, the wound itself secretes a variety of chemokines which recruit stem cells from the circulation, mobilized from the bone marrow, to participate in the formation of blood vessels, and transplanted BMMSCs can be used to meet this demand [78]. Evidence indicates that the paracrine effect of growth factors is the most important mechanism by which MSCs repair tissues. Among these growth factors, VEGF is an important factor that promotes vascular regeneration, which plays an important role in wound healing. Published studies have suggested that transplanted BMMSCs can mobilize within the body and produce VEGF and that they can also function via paracrine effects to complete transformation and tissue repair and promote healing in diabetic ulcers [28]. BMMSCs themselves can also secrete VEGF to induce angiogenesis and thereby bring adequate nutrition to the wound repair area and remove local metabolites. In addition, other factors are involved in this process, including insulin-like growth factor 1 (IGF-1), epidermal growth factor (EGF), keratinocyte growth factor (KGF), stromal cell-derived factor 1 (SDF-1), macrophage inflammatory protein 1a, MMPs, liver cell growth factor (HGF), and erythropoietin (EPO) [26–37]. Studies have specifically shown that BMMSC transplantation can inhibit pFAK and increase the levels of EGF and IGF-1 [79]. Research has also shown that the mechanisms by which BMMSC transplantation acts to effectively promote ulcer healing include increasing the levels of endothelial growth factor (EGF), intracellular adhesion molecule 1 (ICAM-1), and vascular cell adhesion molecule 1 (VCAM-1) and activating the Akt signaling pathway [22].

### 4.2. Umbilical Cord Blood Mesenchymal Stem Cell Transplantation and Diabetic Wounds

Although umbilical cord blood MSCs are an additional rich source of stem cells which is not associated with ethical issues or immunological risk, a number of studies have suggested that these cells promote adverse effects, including myocardial infarction and lung injury [80–82]. Other studies have shown that transplantation of umbilical cord blood MSCs into diabetic foot ulcers significantly promotes the healing of ulcers and that the mechanism involved in this process is the same as that in BMMSC transplantation. Beyond these studies, many other studies have suggested that transplanting human umbilical cord blood stem cells (HUCBSCs) promotes the healing of diabetic wounds in rats via a mechanism involving promotion of the release of keratin 19 from keratinocytes, which in turn promotes the formation of ECM [45]. Certain studies have also noted that transplanting umbilical cord blood stem cells to promote wound healing in diabetic animals is associated with TGF-*β* signaling [83]. Other studies have found that umbilical cord MSC transplantation can increase the levels of VEGF, platelet-derived growth factor (PDGF), and KGF and promote wound healing in diabetic mice [84]. In experiments conducted by Elsharawy and colleagues, rats were randomly divided into three groups: a control group, a diabetic group, and a stem cell-treated diabetic group. The researchers used immunomagnetic beads to obtain human umbilical cord blood CD34+ hematopoietic stem cells, which were then transplanted into the treatment group. They found that CD34+ stem cells have the ability to both differentiate into VECs, which can promote angiogenesis, and act in a paracrine manner to stimulate the formation of new blood vessels to aid the formation new keratinocyte- and dermal fibroblast-mediated collagen deposition and to promote wound healing. These data indicate a new method for treating diabetic wounds [85]. However, in addition to the risks involved in stem cell transplantation, there are ethical issues to consider. Studies have suggested that the mechanism by which transplanted HUCBSCs promote healing in diabetic mice is promotion of the secretion of nerve growth factor (NGF), which can in turn promote the regeneration of both nerve fibers and blood vessels [86].

Li et al. transplanted human umbilical cord blood MSCs to treat diabetic foot ulcers and evaluated the distribution of Treg/Th17/Th1 cells, Treg/Th1 cells, Th17/Th1 cells, and Treg/Th17 cells. They also analyzed a variety of cytokines and other factors (FoxP3, IL-17, IFN-*γ*, TNF-*α*, C-RP, VEGF, Th17/Th1, and CD4+/CD25hi/FoxP3+ cells) and found that Treg/Th17, CD4+/CD25hi/FoxP3+, and Treg/Th1 cells were significantly increased. Moreover, Treg cells were the main functional type of cell in umbilical cord blood MSC transplantation used to treat type 2 diabetic foot ulcers. These cells secreted VEGF and bFGF to improve ulcer healing [87].

### 4.3. Adipose Mesenchymal Stem Cell Transplantation and Diabetic Wounds

Adipose MSCs are a rich source of stem cells which can be obtained with low risk. Studies have suggested that, in diabetic mice transplanted with adipose MSCs, ulcer healing was significantly improved, even though the local blood vessel density and length were not increased via inhibition of the proliferation of fibers [88]. Another animal experiment confirmed that adipose MSC transplantation contributed to the healing of skin ulcers in diabetic mice [89]. That study also suggested that adipose MSCs that were isolated using a micronized cellular adipose matrix (MCAM) injectable method effectively improved wound healing in diabetic skin ulcers. This technique therefore provides a simple, safe, and minimally invasive method to induce tissue repair with the aim of healing ischemic diabetic ulcers [90]. The authors also declared that adipose-derived MSC transplantation may improve lower extremity percutaneous oxygen partial pressure and promote local ulcer healing in both diabetic and nondiabetic lower limb ischemia, which demonstrates that this therapy is equally useful in these two conditions [63].

In summary, wound healing processes are complex. They include vascular closure; the formation of blood clots and acute inflammatory reactions; cell migration, proliferation, and differentiation; angiogenesis; the formation of epithelium; the synthesis of ECM; and reconstruction of the vasculature [85]. Granulation and epithelial metaplasia are the most important mechanisms by which chronic ulcers are healed. Studies have suggested that diabetic chronic wounds are difficult to heal mainly as a result of a lack of an adequate blood supply [91]. BMMSCs are able to secrete soluble factors, such as VEGF, which is one of the strongest proangiogenic factors and a stimulator of the formation of granulation tissue and epithelial cells, keratinocytes, and fibroblasts. BMMSCs can migrate into the trauma area or into inflammatory lesions, where they can stimulate cell proliferation and differentiation and promote the repair of damaged tissue by inducing proliferation and differentiation through processes that involve growth factors, matrix remodeling, immune regulation and anti-inflammatory activities. However, a problem lies in the fact that, according to the investigations of Mishra et al. and Tolar et al. [92, 93], BMMSCs can differentiate into cancer-related muscle fibroblasts and sarcomas. We should therefore pay attention to the safety of stem cell therapy in clinical applications.

## 5. Mesenchymal Stem Cell Transplantation and Diabetic Peripheral Neuropathy

Diabetic peripheral neuropathy is a common complication of diabetes, hyperglycemia, hyperlipidemia, and other metabolic disorders, which increases oxidative stress and induces endothelial dysfunction. These lead to microvascular ischemia and nutritional disorders of the nervous system, which are the underlying pathophysiological basis of the disease [94]. Aldose reductase and *α*-lipoic acid may provide certain beneficial effects, but intensive control of blood glucose cannot completely reverse the progression of the disorder. There is currently no effective treatment for this condition [95, 96]. In recent years, however, several researchers have noted that cell therapy may be a promising method for treating diabetic peripheral neuropathy [97].

### 5.1. Bone Marrow Mesenchymal Stem Cell Transplantation and Diabetic Peripheral Neuropathy

Shibata et al. [98] transplanted BMMSCs, which had been isolated from diabetic rats, into the skeletal muscles of the lower extremities of diabetic rats. At 4 weeks after transplantation, the results suggested that transplantation of BMMSCs increased the expression of VEGF and bFGF in the lower limb muscles and improved peripheral neuropathy in the diabetic rats. It was proposed that the mechanism for this activity was the BMMSCs' multipotent differentiation capabilities and secretion of angiogenic factors, such as VEGF and bFGF, to improve the microenvironment. In particular, these cells have the potential to differentiate into neural cells, including astrocytes and, to a lesser extent, oligodendrocytes and Schwann cells. They may also differentiate into vascular structures and have beneficial effects in diabetic peripheral neuropathy. Kim et al. transplanted BMMSCs into diabetic mice via sciatic nerve injections. At 2 weeks after transplantation, they found that the expression of certain neurotrophic factors, such as NT3 and NGF, was increased, but this effect gradually disappeared after four weeks [99]. We can therefore conclude that BMMSCs exert their effect only in 2–4 weeks after transplantation. Because these two studies involved allograft transplantation, it is possible that the immune responses of the cells caused the disappearance of the treatment effect. In addition, tumor responses have been observed after BMMSCs were transplanted into diabetic mice [40]. Hence, the safety of this transplantation must be further analyzed, and more clinical studies are needed to confirm its efficacy.

### 5.2. Human Umbilical Cord Mesenchymal Stem Cell Transplantation and Diabetic Peripheral Neuropathy

Naruse et al. randomly divided male immune-deficient mice (rnu/rnu F344/N) into a control group and a diabetic group. The diabetic model was induced via an intraperitoneal injection of streptozocin (STZ), and HUCBSC transplantation was performed via an intramuscular injection. Saline was injected in the control group. The results suggested that, in the diabetic group, the unilateral muscle into which the cells were transplanted showed significantly improved damage. In contrast, in the control group, there was no significant difference between the limbs. Histological analysis showed that, on the HUCBSC-injected side, the number of microvessels in the hind limb skeletal muscle in the diabetic group was higher after transplantation than the number in the control group. In the control group, again, there was no significant difference between the limbs [100]. These findings indicate that transplantation of HUCBSCs avoided adverse immune responses and induced neovascularization in and increased the blood supply to the ischemic area. As HUCBSCs have a higher proliferative capacity than other types of stem cells, they may be a more promising treatment option for patients with diabetic peripheral neuropathy.

At the present time, several mechanisms have been proposed to contribute to diabetic peripheral neuropathy, including ischemia and hypoxia, altered nerve polyol metabolism, a decrease in neurotrophic factors, ROS formation, and nervous system-related microvascular diseases. Studies have shown that transplantation of HUCBSCs promoted healing in diabetic rats and that this mechanism also promoted angiogenesis and neural regeneration, increased the survival time of nerves, and increased the secretion of neurotrophic factors, all of which contribute to treating diabetic neuropathy [86].

### 5.3. Adipose Mesenchymal Stem Cell Transplantation and Diabetic Peripheral Neuropathy

Compared to other stem cell sources, adipose MSCs have the following significant advantages: (1) they require only minimally invasive surgery; (2) the cells are located in mature mesenchymal tissue; (3) the process does not require* in vitro* cultures; (4) there is a low immunogenicity risk; and (5) this procedure has high degree of commercialization and is thus convenient to perform, and because it does not require* in vitro* culture, the risk of chromosomal aberration is eliminated [101]. Adipose MSCs have been used to treat a variety of diseases. The mechanisms by which these cells act include homing to the target cell and the release of neurotrophic factors (e.g., epidermal growth factor, TGF-*β*, VEGF, bFGF, HGF, IGF-1, and bone-derived growth factor (BDGF)) which are lacking in diabetic peripheral neuropathy. In addition, these cells regulate immune functions in the same manner as other stem cells [69, 70, 73]. One pilot investigation indicated that autologous transplantation of adipose-derived MSCs improved peripheral neuropathy in diabetic patients with lower limb ischemia. Other studies have suggested that the differentiation and proliferative abilities of autologous ASCs which are isolated from patients with chronic ischemic complications are more limited than those of cells from healthy individuals, so these cells may not be an ideal stem cell source. Hence, further animal experiments and clinical studies are required to explore these issues [102].

## 6. Ideas for Improving the Efficacy and Therapeutic Effects of Stem Cell Transplantation

To improve the therapeutic effects of stem cell transplantation, it is necessary to ensure that the active stem cells directly affect the lesion. In practice, stem cells may sometimes fail to reach the lesion and may at times be killed by chronic inflammation. Studies have indicated that the ability of proliferation to promote angiogenesis affected the degree of the wound healing efficiency of autologous stem cell transplantation [103]. Therefore, it is necessary to determine what factors affect the efficiency of autologous stem cell transplantation in diabetic patients and to explore how stem cells can be modified to improve the efficiency of the transplantation [104].

Meng et al. found that transplantation of BMMSCs in diabetic mice in which osteopontin had been knocked out significantly shortened healing times in comparison to the healing times in normal diabetic mice, indicating that osteopontin is a key regulator of transplanted BMMSCs in mice with diabetic ulcers [105]. Using BMMSC-conditioned medium, Li et al. found that these cells generated reduced levels of ROS and that the phosphorylation of MEK1/2 and ERK1/2 was increased; these effects may be involved in promoting healing in diabetic ulcers [106]. In another study, ethanol extract obtained from the bark of* Mallotus philippinensis* improved MSC proliferation and transplantation efficiency [107]. BMMSC transplantation, when combined with autologous platelet gels, also significantly improved wound healing efficiency [108]. As we have previously stated, overexpression of PGC-1*α* in BMMSCs reduced cell apoptosis and increased ulcer healing efficiency after transplantation, and these effects were also observed in adipose MSC transplantation [20, 59]. Cotransplantation of 14S, 21R-diHDHA, and BMMSCs further improved the secretion of VEGF in the wound area and promoted reconstruction of the local microcirculation and wound healing in diabetic mice [109]. Overexpression of EGF also increased the ICAM-1- and VCAM-1-mediated adhesion reactions and activated the Akt signaling pathway, which promoted the formation of blood vessels and reconstruction of the local circulation to achieve better wound healing [22]. In one study, the survival rate of BMMSCs was increased by hypoxic preconditioning, which increased autophagy to promote angiogenesis and significantly reduced cell apoptosis [110]. In another study, researchers compared standard adipose-derived MSCs to adipose-derived MSCs which were cultured in an anoxic environment* in vitro*. Both cell groups were derived from the same donor. The researchers found that the cells in the two groups were essentially consistent in their expression of surface markers but that telomerase activity was increased in the adipose-derived MSCs that were cultured in an anoxic environment and that these cells had an increased potential to differentiate into adipose cells and osteoblasts [111]. On one hand, these results indicate that the mechanism by which hypoxic preconditioning affects stem cells is the promotion of proliferation and the cells' ability to differentiate; on the other hand, a variety of cells can be used to increase the telomerase activity of subunit h-TERT to achieve improved efficiency in stem cell transplantation [112]. The proliferation and differentiation of MSCs can also be improved by using local infrared irradiation [113]. Recent studies have additionally shown that using zoledronate inhibits mTOR, reduces DNA damage, and reduces apoptosis in BMMSCs in mice [114].

Although using stem cell transplantation to treat the diabetic foot has achieved certain success in clinical trials, optimizing this platform as a cell therapy is still very important, and MSCs have shown great potential for use in tissue engineering as well. Identifying methods to achieve better transplantation efficiency is a constant goal of the researchers exploring this technology. Biomimetic matrices and engraftation can provide stem cells with structural supports which aid in their attachment, proliferation, and differentiation. The local use of silk fibroin scaffold proteins in particular has been shown to increase the ability of endothelial cells to migrate and to secrete proangiogenic factors, improving the usefulness of adipose MSCs in treatments aimed at healing diabetic mouse skin ulcers [115]. Researchers can also choose from many other scaffolds that support MSCs [116–119].

The choice of stem cell delivery system is also very important. The perfect stem cell delivery system provides the best support for stem cell adhesion, proliferation, and differentiation. Hydrogel is one of the most commonly used delivery systems, providing cells with a three-dimensional insoluble reticular structure that can retain water and support cellular activities [120] and can support the adhesion, proliferation, and differentiation of stem cells [121]. Specifically, PEG hydrogel can be used to maintain the activity of stem cells [122]. Prussian blue polymer gel has an anti-ROS effect, and it reduces apoptosis in transplanted stem cells [123]. Additionally, fiber proteins have good biocompatibility and biodegradation properties, and they are essential during wound healing processes; therefore, the development of a fibrin-based cell injection delivery system has shown promise. One study found that the cell transfer efficiency and stem cell proliferative ability of fibrin were dependent on the concentration of fibrinogen and thrombin in the solution used. It is therefore necessary to pay more attention to these factors [124]. Bensaïd et al. reported that fibrin scaffolds that activate fibrinogen and thrombin also promote the efficiency of MSC proliferation and migration [125]. Fibrin glue, which is made of thrombin and fibrinogen combined with PDGF, effectively stimulates the effects of MSC transplantation therapies [126]. Studies have also suggested that a collagen scaffold and a fibrin spray system could be used to improve the efficiency of BMMSC transplantation and to significantly improve the healing of ulcers in diabetic mice, respectively [14, 127]. Another study has shown that BMMSCs that were locally injected and then covered with an autologous skin collagen membrane significantly improved the rate of healing in skin ulcers in diabetic patients [128]. Enriching a collagen matrix with adipose MSCs in a silicon membrane also improved the efficiency of ulcer healing in diabetic mice [129]. In yet another study, Jiang et al. invented a carrier coated with an acrylic film via plasma polymerization to transfer adipose tissue-derived MSCs to the local wound area and showed that it significantly reduced the effect of TNF-*α* on the inflammatory response; increased the activation of anti-inflammatory M2 macrophages, which are induced by TGF-*β*-dependent angiogenesis; clearly increased the formation of differentiated fiber cells; and improved the efficiency of tissue repair [73, 130]. In addition, three-dimensional cultures of adipose MSCs increased HGF secretion, which may improve transplantation efficiency [61].

## 7. The Role of Mesenchymal Stem Cell-Derived Exosomes and MicroRNAs in Diabetic Wound Healing

In 2010, Lai et al. found that the cell membrane can secrete 40–100 nm vesicles under physiological or pathological conditions; these so-called exosomes can contain many proteins and RNAs. These exosomes form through membrane fusion and endocytosis to transport biological activators to target cells, thus affecting the target cells, allowing the exchange of information between cells and promoting tissue repair as well as regulating immune function [131]. The function of exosomes is based on the type of cell from which they originate, and MSCs are the richest source of exosomes [132]. We now review the role of MSC-derived exosomes (MEX) in tissue rehabilitation and wound healing.

### 7.1. RNA-Mediated Damage Rehabilitation

#### 7.1.1. RNA

Researchers have indicated that the mRNA in MEX plays a role in the repair of tissue damage. Bruno et al. [133, 134] specifically revealed that mRNA in MEX is mainly associated with the promotion of proliferation, transcription regulation, and immune regulation and plays a role in the repair of renal injury. Gatti et al. [135] declared that, in acute kidney injury induced in rats by ischemia/reperfusion, the mRNA in MEX can promote the proliferation and inhibit the apoptosis of the renal tubular epithelial cells. Zhu et al. [136] found that MEX contain mRNA encoding KGF, can transfer material from BMMSCs to type II epithelial cells, transfect cells with the KGF protein, inhibit the immune response, and reduce lung tissue damage. However, as few studies have studied the effects of the proteins in MEX on diabetic wound healing, further investigations are still needed.

#### 7.1.2. MicroRNA

MicroRNA (miRNA) is a type of noncoding single-stranded RNA which is encoded by endogenous genes. miRNA is involved in the regulation of posttranscriptional gene expression and plays an important role in wound rehabilitation [137]. Many researchers have declared that the main function of miRNAs such as miR-21, miR-27b, miR-95, miR-203, and miR-210 is to regulate posttranscriptional gene expression by binding to target mRNAs, leading to mRNA degradation, suppression of translation, or even gene activation. Thus, miRNAs are promising therapeutic targets and demonstrate great potential as diagnostic biomarkers for diabetic wound healing [138, 139]. The use of miRNAs to treat diabetic wounds has undergone many advancements; for example, the upregulation of both miR-21 and miR-126 enhances wound healing by stimulating leukocyte migration to the wound site and promoting bacterial control and wound closure through enhancement of keratinocyte and fibroblast activation and migration and improved angiogenesis. The downregulation of miR-203 and miR-210 also enhances wound healing by promoting keratinocyte migration [140–142].

miRNAs can induce the secretion of many cytokines, such as TNF-*α*, IL-6, and IL-1*β*, and can regulate many signaling pathways, such as the PI3 K-Akt, mTOR, Toll-like receptor (TLR), NF-*κ*B, and IGF-1 pathways. These factors can regulate inflammation and cell proliferation and maturation, leading to the healing of diabetic wounds [143–147]. These factors can also reconstruct the microcirculation and relieve diabetic ischemic complications such as PAD to further help in the healing of diabetic wounds [148–150].

MEX contain a large amount of miRNA. Research has found that, in rats with arterial embolization in the brain, miR-133b in MEX can be transferred from BMMSCs to damaged neurons, regulating tyrosine hydroxylase and dopamine transporters, inducing neurite growth, and promoting rehabilitation of the injured brain tissue [151]. In addition, Bonafede et al. [152] constructed an* in vitro* cell model of amyotrophic lateral sclerosis and discovered that exosomes from ASCs can have a neuroprotective effect on this condition. The mechanism is thought to be the secretion of miRNAs, such as miR-21 and miR-222, to promote cell proliferation to in turn induce tissue rehabilitation. Feng et al. [153] found that the level of methylation of CpG binding protein 2 was increased in the myocardium after myocardial ischemia and that miR-22 can specifically affect the methylation of CpG binding protein 2, reducing cell apoptosis. miR-19a delivered by BMMSC-derived exosomes has similar effects, and the mechanism may lie in inhibition of the expression of PTEN and BIM protein and activation of the Akt and ERK signaling pathways [154]. Nakamura et al. used an intramuscular injection of MEX in a model of muscle damage and discovered that it could induce muscle cell proliferation, migration, and angiogenesis and repair muscle damage and that these effects were associated with miR-494 [155]. Recently, Wang et al. were the first to find that miR-223 in MEX has cardioprotective effects in sepsis, providing a new approach to the treatment of sepsis [156]. Lipopolysaccharide-preconditioned MSCs may also have improved abilities to regulate macrophage polarization and resolve chronic inflammation by shuttling let-7b, and exosomes derived from these cells can activate TLR4/NF-*κ*B/STAT3/Akt and enhance diabetic cutaneous wound healing [157].

In sum, as a good transport carrier, MEX can play an important role in transporting miRNA in the process of tissue rehabilitation. However, further exploration of the application of MEX in diabetic foot ulcers and their vascular complications is needed.

### 7.2. Protein-Mediated Damage Rehabilitation

The biological function of proteins contained in exosomes was first confirmed in immunological research [158], but, until 2010, the damage rehabilitation function of MEX proteins had not been reported [159]. This rehabilitation mainly occurs through the regulation of intracellular signaling pathways to promote cell survival and proliferation, reduce cell apoptosis, and affect a variety of different tissue repair processes. Research has found that MEX proteins can also increase ATP and NADH-I levels, decrease the phosphorylation of c-JNK, reduce oxidative stress, and activate the PI3 K/Akt pathway to promote cell proliferation, which all play an important role in tissue repair, but the specific proteins involved still need further research [159–161]. Zhang et al. revealed that human umbilical cord MEX can transfer Wnt4 protein to activate endothelial cell *β*-catenin and its subsequent signal pathway, in addition to enhancing angiogenesis and promoting the rehabilitation of skin lesions [162]. In the nervous system, Katsuda et al. found that ASC-derived exosomes contain neprilysin which is transported to Neuro-2a cells to induce overexpression of amyloid precursor protein and degradation of amyloid *β* protein, resulting in decreased levels of this protein [163]. Regarding immune regulation, research has also found therapeutic potential for the enzymes transported by MEX in graft-versus-host disease [164]. Although a few studies of the effects of the proteins in MEX on diabetic wound healing have been conducted, further investigations are still needed.

Based on a large number of studies in a variety of animal models, it has been suggested that MEX rehabilitate damage. The RNAs and proteins contained in the exosomes can be delivered to target cells, so MEX are considered to be the ideal drug delivery system [165]. In addition, studies suggested that pretreatment of target cells, such as by gene editing, can change the secretion characteristics and function of the exosomes. It is believed that, after pretreatment, MSCs may be the ideal drug or gene delivery media [166, 167]. In addition, MEX in different growth stages or culture media contain different RNAs and proteins, so the processes of extraction and collection also need to be standardized [168, 169].

The function of MEX also has individual differences; in particular, different sources of MSCs may yield MSCs which produce different cytokines and have varying abilities to respond to inflammation [170]. Phenotype, donor age, and gender may also affect the features of MSCs, such as surface markers and the ability to clone them [171]. However, we need to further clarify the relationship between the MSC donor and the secretion function of exosomes as well as the relationship between the therapeutic effect and bioactive substances to provide more accurate information prior to the clinical application of MEX in damage rehabilitation.

## 8. Summary and Perspective

**Figure 1:**
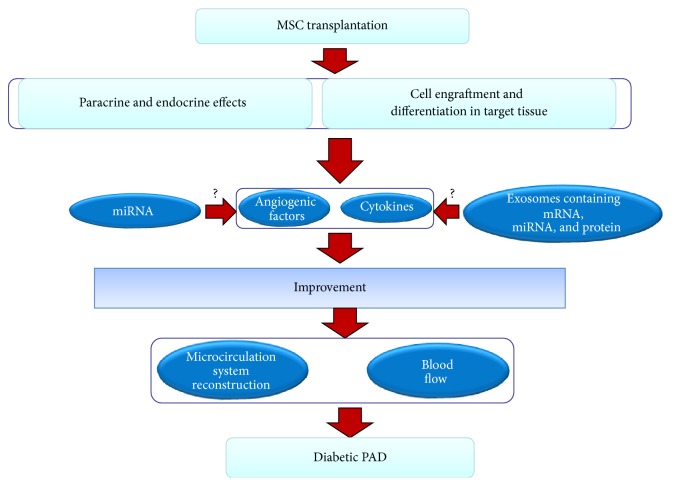
Mechanism of the effect of transplantation of MSCs on diabetic PAD. The mechanism of the recovery effect mediated by stem cell transplantation stems from two pathways: one is the secretion of angiogenic factors and cytokines and the other is the engraftment and differentiation of the cells into tissue constituents. Stem cells can specifically improve the local secretion and expression of angiogenic factors and cytokines, which contributes to the reconstruction of the microcirculation system and the improvement of blood flow and islet *β* cell dysfunction, in turn leading to the improvement of diabetic PAD. Stem cells can also differentiate into endothelial cells to achieve recovery of endothelial cell dysfunction. These effects may be associated with miRNA and MEX.

**Figure 2:**
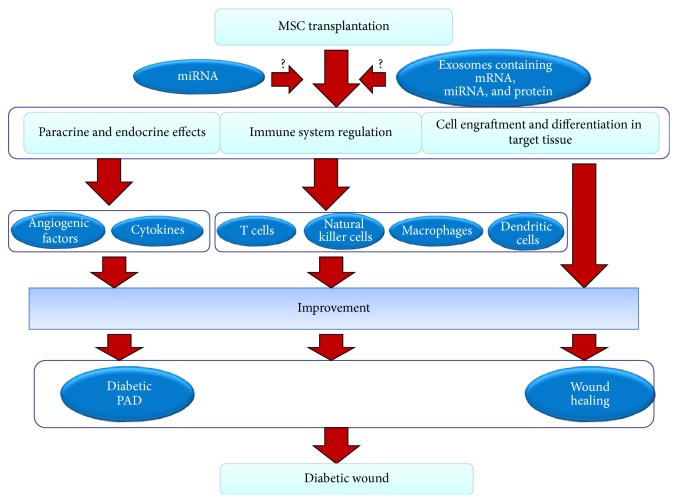
Mechanism of the effect of transplantation of MSCs on diabetic wound. The mechanism of diabetic wound recovery by MSC transplantation stems from three pathways: the first is the secretion of angiogenic and factors and cytokines, the second is regulation of the immune system, and the third is the engraftment and differentiation of the cells into tissue constituents. Stem cells can specifically improve the local secretion and expression of angiogenic factors and cytokines, which contributes to the improvement of diabetic PAD and diabetes itself. Stem cells can also regulate the activities of T cells, natural killer cells, macrophages, and dendritic cells and can inhibit infections and inflammatory reactions. Moreover, MSCs can differentiate into target tissues to achieve a reparative effect. These effects may be associated with miRNA and MEX.

**Figure 3:**
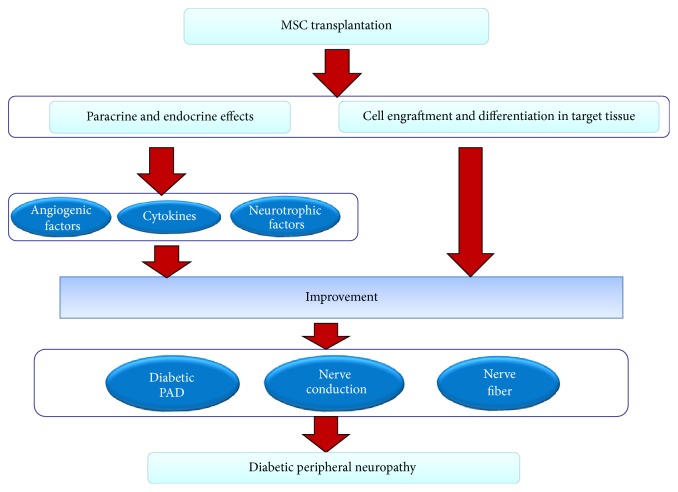
Mechanism of the effect of transplantation of MSCs on diabetic neuropathy. The mechanism of the recovery effect mediated by stem cell transplantation stems from two pathways: one is the secretion of angiogenic factors, cytokines, and neurotrophic factors and the other is the engraftment and differentiation of the cells into tissue constituents. Stem cells can specifically improve the local secretion and expression of angiogenic factors and cytokines, which contributes to the improvement of diabetic PAD and diabetes itself, in turn leading to the improvement of diabetic neuropathy. Neurotrophic factors can also ameliorate the dysfunction of nerve fibers and nerve conduction. Moreover, stem cells can differentiate into the target tissues to achieve a reparative effect.

In summary, MSC transplantation is a new technology that can be used to treat the diabetic foot and is a well-studied topic in the field of angiogenesis. MSCs have high proliferative and self-renewal capabilities in addition to the ability to differentiate into multiple types of cells, including VECs, SMCs, and astrocytes and, to a lesser extent, oligodendrocytes and Schwann cells, after transplantation. The transplanted stem cells regulate the immune system by influencing the immune responses of T cells, natural killer cells, macrophages, and dendritic cells, and they participate in diabetic wound healing. Via both endocrine and paracrine effects and the secretion of angiogenic factors, cytokines and neurotrophic factors that promote angiogenesis, the blood flow in the local tissue recovers, and neurological lesions are healed. MEX also participate in the wound healing process via the effects of the mRNA, miRNA, and protein molecules which they contain (Figures 1, 2, and 3). Although certain researchers argue that transplanted MSCs can also recover islet *β* cell dysfunction and maintain balanced blood glucose levels, these phenomena seem to lack supporting evidence [172]. In animals with diabetic feet and in clinical trials, the transplantation of MSCs has led to positive results, and, in short-term follow-ups, there have been no significant adverse reactions or serious complications. The MSC transplantation technique has therefore been successfully developed, and it provides a basis for clinical applications involving stem cell transplantation to treat the diabetic foot.

In recent years, a large number of* in vivo* animal models have been used to study the idea that transplanted MSCs promote local blood vessel formation. The transplanted MSCs secrete a large number of cytokines and growth factors under certain conditions, participate in the formation of new capillaries, improve the local microcirculation, increase the blood supply to peripheral blood vessels, and promote healing in the diabetic foot. This transplantation is therefore one of the prospective treatments for diabetic lower limb ischemia, diabetic ulcers, and diabetic peripheral neuropathy, and it has become an important topic of research in the life sciences. However, the following problems remain: (1) methods to obtain more MSCs are needed; (2) the purity of MSCs must be improved; (3) stem cell quality detection methods must be unified; (4) the method to achieve the best therapeutic effect using MSCs is yet to be determined; (5) methods to identify the survival rate, effectiveness, and long-term efficacy of MSCs after transplantation are needed; (6) after transplantation, the possibility of uncontrolled cell differentiation and proliferation and the development of tumors must be explored; and (7) the detailed mechanism by which MSC transplantation functions as a treatment for the diabetic foot remains unknown. We believe that these problems will be solved gradually in future investigations.
